# Cardiac Metastasis From Lung Cancer: A Hidden Cardioembolic Source of Acute Ischemic Stroke

**DOI:** 10.7759/cureus.116107

**Published:** 2026-09-11

**Authors:** Gabriela Paulo, Ilda Coelho, Alexandra Sousa, Márcia Ribeiro

**Affiliations:** 1 Department of Internal Medicine, Unidade Local de Saude de Barcelos/Esposende, Barcelos, PRT; 2 Department of Cardiology, Unidade Local de Saude de Barcelos/Esposende, Barcelos, PRT

**Keywords:** acute ischemic stroke, cancer-associated stroke, cardiac metastasis, cardioembolic stroke, non-small cell lung cancer

## Abstract

Acute ischemic stroke requires prompt identification of the underlying mechanism to guide treatment and secondary prevention. Although common causes account for most cases, occasionally the diagnostic work-up reveals an unexpected cardioembolic source. We report the case of a woman in her 50s with stage IVB non-small cell lung carcinoma who was admitted with presyncope, acute disorientation, and dysarthria, followed by anomia and right-sided neurological deficits. Brain magnetic resonance imaging revealed multiple acute ischemic infarcts in the left middle cerebral artery territory. Cardiac imaging identified a left ventricular mass highly suggestive of cardiac metastasis, considering the probable source of cerebral embolism. Despite treatment, her condition progressively deteriorated, and she died one year later. This case highlights cardiac metastasis as a rare but important cause of cardioembolic stroke in patients with active cancer and emphasizes the value of multimodal cardiac imaging in identifying unusual embolic sources.

## Introduction

Lung cancer is the most common malignancy worldwide and is responsible for the highest number of cancer-related deaths [1]. Owing to its often asymptomatic early course, a substantial proportion of patients present with advanced disease at diagnosis, with nearly half presenting with distant metastases [2]. The brain, bones, liver, and adrenal glands are the most frequent metastatic sites [3], whereas cardiac involvement is rare and usually reflects disseminated disease [4,5].

We present a patient with advanced non-small cell lung cancer who developed acute ischemic stroke secondary to an uncommon cardiac source of embolism. This case highlights the rare occurrence of cardiac metastasis and the clinical challenge of identifying an unusual cardioembolic source in patients with active malignancy.

## Case presentation

A woman in her 50s presented to the Emergency Department with presyncope. On examination, she was hemodynamically stable, afebrile, and eupneic, with an oxygen saturation of 97% on room air. The lungs were clear to auscultation. Cardiac auscultation revealed a regular rhythm with no murmurs. There was no jugular venous distension or peripheral edema. She was disoriented and exhibited mild dysarthria, with no other focal deficits.

Two months earlier, she had been diagnosed with stage IVB (cT4N2M1c) non-small cell lung carcinoma (NSCLC), with metastases to the L1 vertebral body and the inferior phrenic lymph node. Histopathological analysis demonstrated adenosquamous carcinoma (thyroid transcription factor-1 (TTF-1) negative) from the basal bronchus and adenocarcinoma (TTF-1 positive) from the main bronchus. Molecular profiling showed high programmed death-ligand 1 expression (90%-100%) and an exon 20 mutation on next-generation sequencing. She had completed palliative radiotherapy to the L1 vertebral metastasis and was awaiting initiation of systemic therapy while eligibility for a clinical trial investigating targeted therapy was being assessed. Her regular medication included transdermal fentanyl 25 µg/hour and immediate-release morphine 10 mg three times daily, the latter introduced one week earlier. She reported no history of tobacco or alcohol use.

Cranial computed tomography showed a 6-mm, contrast-enhancing lesion in the head of the left caudate nucleus, slightly protruding into the adjacent ventricle without edema, suggestive of a metastatic lesion (Figure 1). Given these findings, brain magnetic resonance imaging (MRI) was subsequently performed, revealing multiple recent ischemic lesions in the left middle cerebral artery (MCA) territory, involving the parietotemporo-insular cortico-subcortical regions and the head of the caudate nucleus. In addition, a small, well-circumscribed, contrast-enhancing lesion, anatomically distinct from the areas of infarction, was identified protruding into the ventricular system, compatible with a metastatic lesion and showing minimal surrounding edema (Figure 2).

**Figure 1:**
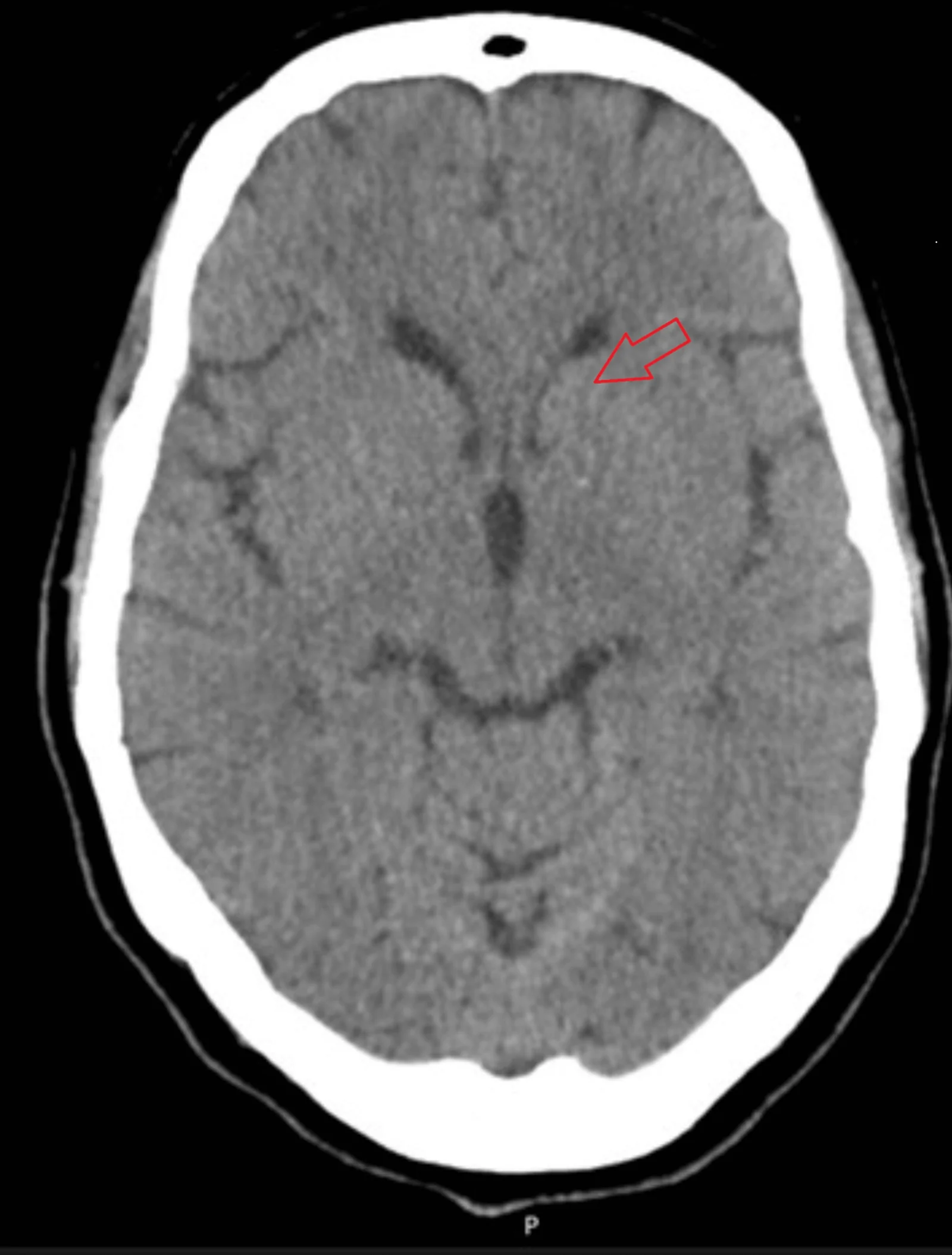
Cranial CT scan Cranial CT demonstrating a 6-mm enhancing lesion in the head of the left caudate nucleus (arrow), with slight protrusion into the adjacent ventricle and no surrounding edema, suggestive of metastatic disease CT: computed tomography

**Figure 2:**
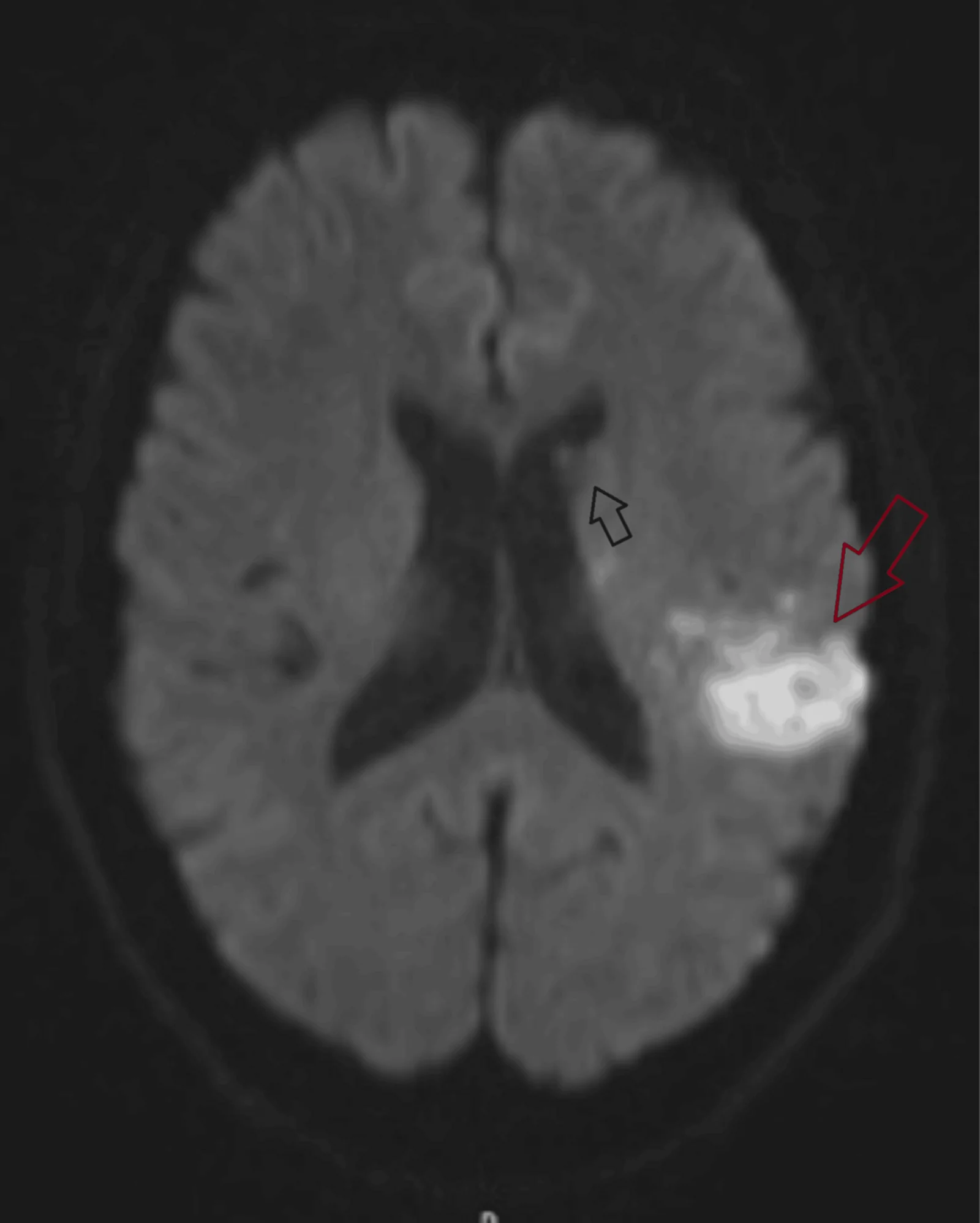
Brain MRI Diffusion-weighted MRI showing multiple recent ischemic lesions in the left middle cerebral artery territory (red arrow) and a small contrast-enhancing lesion protruding into the ventricular system, anatomically distinct from the ischemic lesions (black arrow), compatible with a metastatic lesion MRI: magnetic resonance imaging

Later during her stay in the Emergency Department, the patient experienced acute neurological deterioration, with new-onset focal deficits, including anomic pauses, slight right arm drift on the Mingazzini test, and right-sided hypoesthesia. Her National Institutes of Health Stroke Scale score was 4 [6]. Electrocardiogram showed sinus rhythm, and aspirin was started.

The patient was subsequently admitted for further investigation and clinical monitoring. During hospitalization, transthoracic echocardiography revealed a highly mobile, lobulated, heterogeneous mass measuring approximately 19 × 7 mm within the anterolateral apical segments of the left ventricle. Doppler examination showed no valvular abnormalities. Biventricular systolic function was preserved (Videos 1, 2). Cardiac MRI further characterized the lesion as an intracavitary mass (18 × 12 × 29 mm) arising from the anterior left ventricular wall without a discernible cleavage plane from the adjacent myocardium. The mass showed isointense-to-mildly hyperintense T1 signal, hyperintense T2 signal, slow first-pass perfusion, early contrast enhancement, and diffuse heterogeneous late gadolinium enhancement, findings highly suggestive of a cardiac neoplasm (Figure 3).

**Video 1:** Transthoracic echocardiography (four-chamber view) A highly mobile intracavitary mass is visualized within the apical region of the left ventricle

**Video 2:** Transthoracic echocardiography (four-chamber view with color Doppler) Color Doppler demonstrates preserved intracardiac blood flow without evidence of valvular abnormalities

**Figure 3:**
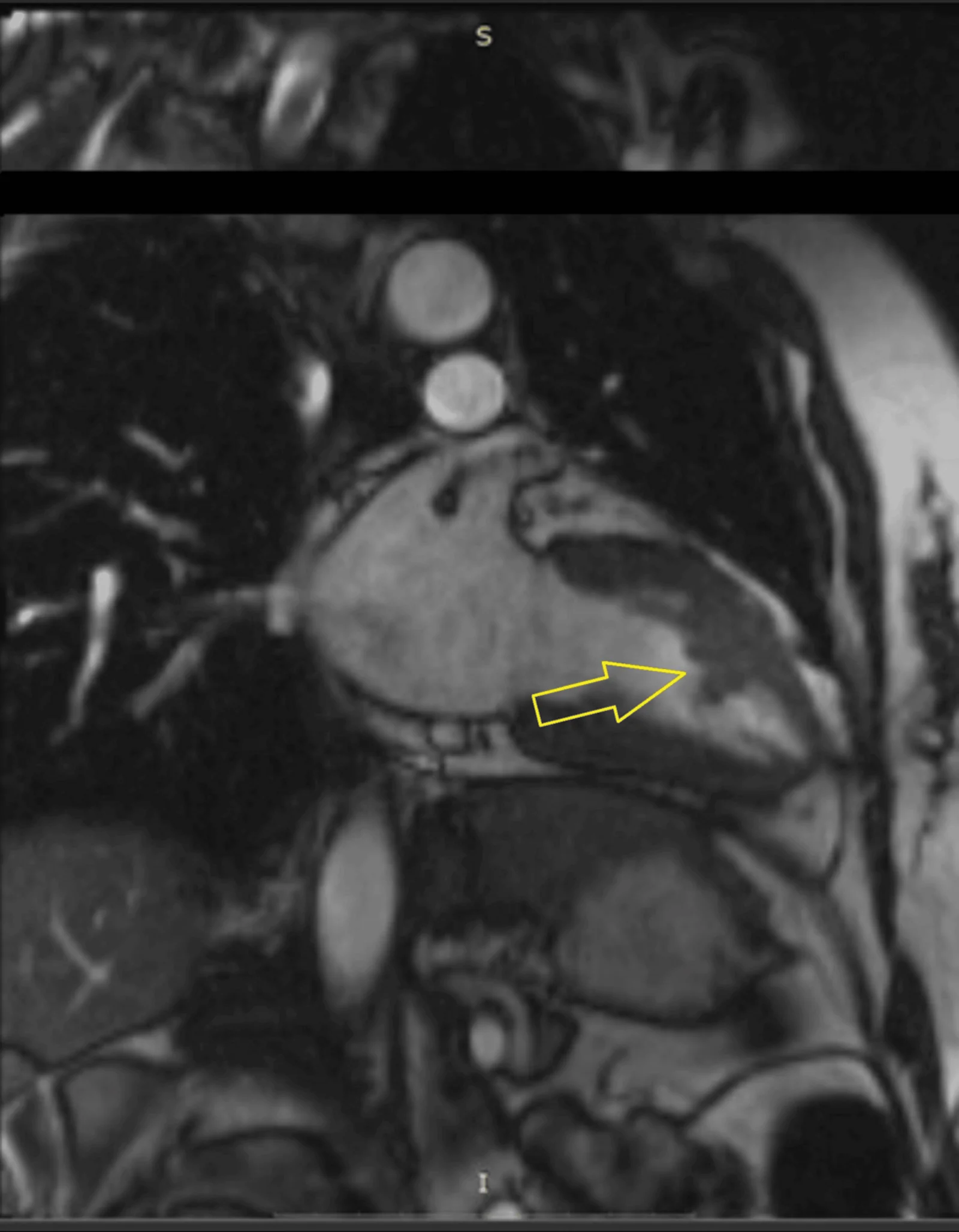
Cardiac magnetic resonance imaging (two-chamber view) Intracavitary mass arising from the anterior wall of the left ventricle (yellow arrow), with no clear cleavage plane from the adjacent myocardium, consistent with a cardiac neoplasm

Given the presence of advanced metastatic NSCLC, the lesion was considered most consistent with a left ventricular metastasis and the presumed source of cerebral embolism. Histological confirmation was therefore not pursued, as an invasive procedure was unlikely to alter management.

Regarding treatment, low-molecular-weight heparin was initiated after aspirin was discontinued. Following consultation with the Oncology team, the patient was deemed ineligible for the planned clinical trial because the tumor histology was not exclusively adenocarcinoma, and systemic chemotherapy with carboplatin and pemetrexed was initiated. Over the ensuing year, her condition progressively deteriorated despite systemic treatment, and she ultimately died from multiorgan failure.

## Discussion

Cardiac tumors are rare, with an estimated incidence of 0.03% [4]. Although uncommon, secondary cardiac involvement is more frequently encountered than primary cardiac neoplasms [4,5]. Available incidence estimates are largely derived from autopsy studies, in which cardiac metastases have been identified in 2.3%-18.3% of cases, with up to 9.1% diagnosed during active disease [4,5]. Among solid tumors, lung cancer represents the most frequent primary source of cardiac metastases, followed by hematological and breast malignancies [4,5].

Several mechanisms may account for cardiac dissemination, including direct invasion, hematogenous spread, and, most commonly, lymphatic extension [5]. Intracavitary extension through major vessels, including the inferior vena cava and pulmonary veins, has also been reported [5]. The resulting clinical manifestations are highly variable and depend on the location and extent of cardiac involvement. Patients may remain asymptomatic or present with hemodynamic compromise, arrhythmias, heart failure, or systemic embolic events, including acute ischemic stroke [4,5].

The association between cancer and cerebrovascular disease is well established. Patients with active malignancy have an approximately twofold increased risk of acute ischemic stroke, particularly within the first year following cancer diagnosis [7]. The risk is especially pronounced in metastatic pulmonary, pancreatic, and gastrointestinal cancers, with adenocarcinoma histology showing a particularly strong association with stroke [7], as observed in the present case with mixed-histology NSCLC containing an adenocarcinoma component.

Although nearly half of acute ischemic strokes occurring in patients with active cancer can be explained by conventional stroke mechanisms (e.g., atrial fibrillation), cancer-specific mechanisms are increasingly recognized as important contributors to cerebrovascular events [7]. According to the 2026 American Heart Association classification framework [7], this case fulfills criteria for probable cancer-related stroke, supported by MRI evidence of left ventricular tumor invasion and multiple cortical infarctions within the left MCA territory. In addition to tumor embolization, other mechanisms associated with cancer-associated stroke include nonbacterial thrombotic endocarditis, paradoxical embolism, and disseminated intravascular coagulation [7].

Cardiac MRI is the reference standard for tissue characterization of cardiac masses, whereas transthoracic echocardiography remains the first-line imaging modality for initial evaluation [4,5]. In our patient, the heterogeneous intracavitary lesion with T2 hyperintensity, contrast perfusion, and heterogeneous late gadolinium enhancement favored a neoplastic over a thrombotic lesion [4,5]. In the context of disseminated malignancy and concordant cardiac imaging findings, histological confirmation was therefore considered unnecessary, as it would have been unlikely to alter management.

Evidence guiding antithrombotic management in cardioembolic stroke associated with intracardiac metastatic disease remains scarce [7]. Nevertheless, anticoagulation was considered appropriate given the presence of a metastatic left ventricular mass and the likely coexistence of a malignancy-associated hypercoagulable state.

Cardiac metastases usually reflect advanced-stage malignancy and are associated with a poor prognosis [4,5]. Survival is largely determined by the underlying primary tumor and the overall metastatic burden [4,5]. In this patient, concomitant involvement of the lumbar vertebrae, brain, and left ventricle reflected widespread systemic dissemination. Adenosquamous histology may have further contributed to the unfavorable outcome, given its association with poorer survival than other NSCLC subtypes [8]. The patient's condition progressively deteriorated despite anticoagulation and systemic therapy, and she died approximately one year after presentation.

## Conclusions

This case highlights the rare occurrence of cardiac metastases, which became clinically apparent following an acute ischemic stroke. During the etiological work-up, transthoracic echocardiography unexpectedly revealed intracardiac lesions, subsequently characterized by cardiac MRI as cardiac metastases, underscoring the pivotal role of multimodality cardiac imaging in the diagnosis and characterization of intracardiac masses. Cardiac metastases are a hallmark of advanced systemic disease and portend a poor prognosis, reinforcing the clinical importance of detecting cardiac involvement, even when clinically unsuspected.
